# Genetic Variability and Aggressiveness of *Stilbocrea banihashemiana*, an Emerging Pathogen Responsible for Cankers of Fig and Fruit Trees

**DOI:** 10.3390/plants15131945

**Published:** 2026-06-24

**Authors:** Zeinab Bolboli, Hamed Negahban, Moslem Jafari, Santa Olga Cacciola, Reza Mostowfizadeh-Ghalamfarsa

**Affiliations:** 1 Department of Plant Protection, School of Agriculture, Shiraz University, Shiraz 7144113131, Iran; zeinabbolboli4@gmail.com (Z.B.); h.negahban@hafez.shirazu.ac.ir (H.N.); 2 Fig Research Station, Fars Agricultural and Natural Resources Research and Education Center, Agricultural Research, Education and Extension Organization (AREEO), Estahban 7451877802, Iran; m.jafary@areeo.ac.ir; 3 Department of Agriculture, Food and Environment (Di3A), University of Catania, 95123 Catania, Italy

**Keywords:** pathogenicity, *Bionectriaceae*, *Ficus carica*, genetic diversity, inter-simple sequence repeats (ISSRs), principal component analysis (PCA), cultivar susceptibility

## Abstract

*Stilbocrea banihashemiana* Bolboli, Tavakolian & Mostowf. is an emerging pathogen causing canker and dieback in a broad range of fruit and ornamental trees in Iran, and its distribution is expanding across the country. Extensive surveys conducted over five consecutive years (2019–2023) yielded 88 isolates of *S. banihashemiana* from multiple hosts, including different fig (*Ficus caricae* L.) cultivars, as well as loquat (*Eryobotria japonica *(Thunb.) Lindl.), pomegranate (*Punica granatum* L.), and walnut (*Juglans regia* L.) trees, across eight distinct regions of southern Iran. Species identification was performed morphologically and molecularly by employing the *S. banihashemiana*-specific primer pair TEF-Sb1 and TEF-Sb3. The genetic diversity of the *S. banihashemiana* population of isolates was assessed using eight inter-simple sequence repeats (ISSRs) markers. The UPGMA dendrogram demonstrated broad genetic variability among the isolates, with similarity coefficient values spanning from 0.46 to 1.00. This wide range indicates the presence of multiple divergent genotypes within the population, rather than a single dominant lineage. Principal coordinate analysis (PCoA) grouped the 88 isolates into three distinct genetic clusters that partially corresponded to geographic origin and host species. Pathogenicity assessment of 53 selected isolates from various hosts and geographic origins on detached fig shoots demonstrated highly significant variability in aggressiveness among isolates originating from different host species and geographically distinct regions. Multivariate analysis using principal component analysis (PCA) combined with heatmap-based clustering of the aggressiveness dataset clearly separated the isolates into four distinct groups, ranging from highly to less aggressive. A susceptibility assessment of 10 fig cultivars using the ex-type-isolate of *S. banihashemiana* revealed that the pathogen caused internal lesions and wood discoloration in all cultivars. Based on statistical analysis, the cultivars were classified into three groups: susceptible (cv. ‘Siah’), moderately susceptible (‘Brown Turkey’, ‘C8-M’, ‘C8-F’, ‘Dehdez’, ‘Gilasi’, ‘Payves’, ‘Shah-Anjeer’ and ‘Sabz’), and less susceptible (‘Matti’). High genetic variability, multiple-host association, and partial geographic structure indicate that in Fars Province *S. banihashemiana*’s population structure and epidemiology are complex, with high adaptive potential. This complexity may influence disease spread, management strategies, and long-term evolutionary trajectories.

## 1. Introduction

*Stilbocrea banihashemiana*, a recently described member of the family *Bionectriaceae*, has been identified as the causal agent of canker and branch dieback in fig and loquat trees [1] and in barberry (*Berberis vulgaris* L.) [2]. The pathogen has also been recovered from symptomatic pomegranate [3] and walnut [4] trees. These studies demonstrated that *S. banihashemiana* possesses a broad host range and can persist in walnut and fig trees as a latent pathogen without inducing visible symptoms, thereby substantially increasing the risk of unnoticed infection. Although the members of *Stilbocrea* have not traditionally been regarded as a major group of canker-causing fungi, increasing evidence indicates that several species are associated with woody plant diseases, including canker, dieback, wood necrosis, and discoloration. Pathogenic species reported from woody hosts include *S. gracilipes* (Tul. & C. Tul.) Samuels & Seifert on pomegranate [5], *S. colubrensis* Lechat & J. Fourn from *Bambusa vulgaris* (Schrad. ex J.C. Wendl.) Nakai [6], and *S. walteri* Voglmayr & Jaklitsch from *Quercus ilex* L. [7], *Citrus aurantifolia* (Christm.) Swingle, *C. aurantium* L., and *C. limon* (L.) Osbeck [8]. Moreover, *S. macrostoma* (Berk. & M.A. Curtis) Höhn is reported to be associated with wood necrosis and discoloration of *Quercus brantii* Lindl. [9]. These findings collectively highlight the emerging phytopathological relevance of *Stilbocrea* species, particularly in woody hosts, especially fruit trees.

The common fig is an economically important fruit crop cultivated extensively in Mediterranean and Middle Eastern regions, including Turkey, Morocco, Greece, Spain, and Iran. According to FAOSTAT [10], annual fig production in Iran exceeds 75,432 tons, with Fars Province representing the principal center of dried fig production due to its favorable agro-climatic conditions [11]. However, during recent decades, the incidence of canker and branch dieback diseases in fig orchards has increased markedly, resulting in substantial economic losses, particularly in rainfed production systems.

Fig canker is recognized as a multifactorial disease complex involving multiple fungal taxa from several ascomycetous families, including *Bionectriaceae*, *Botryosphaeriaceae*, *Ceratocystidaceae*, *Diaporthaceae*, *Diatrypaceae*, and *Nectriaceae* [12,13,14,15,16]. Fig Bionectria canker is typically characterized by extensive wood necrosis, elongated sunken lesions, marginal bark cracking, and occasional cicatricial callus formation [1]. A five-year survey of fruit tree canker diseases in southern Iran resulted in the establishment of a comprehensive collection of *S. banihashemiana* isolates from different fig cultivars, pomegranate, loquat, and walnut trees. Isolates originating from different geographic regions exhibited pronounced phenotypic variability, and both latent and symptomatic infections were observed, providing strong evidence for substantial intraspecific genetic diversity and variability in aggressiveness within *S. banihashemiana* populations.

Understanding the genetic structure of *S. banihashemiana* populations is essential for elucidating the disease epidemiology, investigating reproductive strategies of the fungus, identifying fruit tree cultivars with least susceptibility, and developing sustainable disease management approaches [17,18]. Inter-simple sequence repeat (ISSR) markers are widely used for population genetic studies of phytopathogenic fungi due to their reproducibility, sensitivity, and cost-effectiveness [19,20,21]. In the major fig canker pathogen, *Diaporthe cinerascens* Sacc., ISSR analyses revealed population structuring correlated with geographic origin in southern Iran [22].

The use of resistant cultivars represents the most effective management strategy for fig canker diseases. To date, no effective chemical control measures have been developed, and disease management relies primarily on preventive cultural practices [23]. In Iran, fig cultivar susceptibility has previously been evaluated for Diaporthe canker [13], Botryosphaeria canker [24], and Nectria canker [25], while the cultivar responses to *S. banihashemiana* remain uncharacterized. Accordingly, the objectives of this study were to (i) assess the genetic diversity and population structure of *S. banihashemiana* isolates from different hosts and geographic regions using ISSR markers, (ii) evaluate isolate aggressiveness, and (iii) determine the susceptibility of selected fig cultivars under controlled greenhouse conditions. The results are expected to improve understanding of the epidemiology and evolutionary potential of this emerging pathogen and to support the development of sustainable and integrated management strategies in fig production systems.

## 2. Results

### 2.1. Fungal Isolates

**Figure 1:**
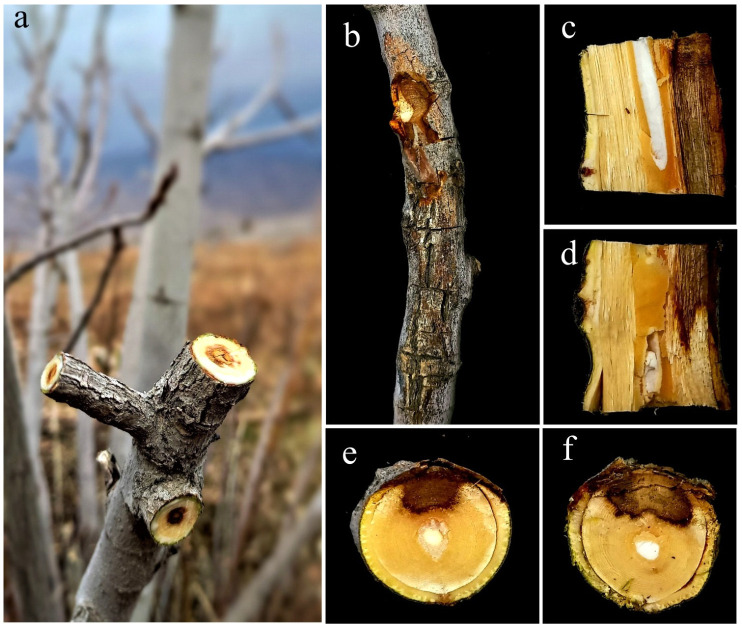
Disease symptoms associated with *Stilbocrea banihashemiana* on infected fig trees in Fars Province, Iran. (**a**,**b**) External bark necrosis accompanied by wood discoloration; (**c**,**d**) longitudinal sections showing wood necrosis and progressive internal discoloration; (**e**,**f**) cross-sections of branch cankers exhibiting bark necrosis and irregular patterns of wood necrosis and discoloration.

A total of 37 fungal isolates were recovered in this study from symptomatic fig trees in commercial orchards of two central districts of Fars Province (Figure 1); they morphologically resembled members of the *Bionectriaceae* family. The colonies exhibited slow growth, forming a cottony mycelial mat that ranged in color from white to grayish-olive green. Microscopic examination revealed abundant conidiogenesis. The conidia were smooth-walled, unicellular, and hyaline, produced on cylindrical, branching phialides, and displayed both globose and allantoid morphologies (Figure S1). Six of these new isolates were obtained from the ‘Sabz’ cultivar in Qalat, while the remaining 31 were collected from various cultivars (‘Sabz’, ‘Shah-Anjeer’, and ‘Kalleh Gorbehie’) within Estahban fig orchards (Table 1; Figure S2). The isolates were subsequently identified at the species level based on molecular analysis (see Section 2.2). Adding these isolates to the 51 previously characterized isolates from the culture collection, a total of 88 *S. banihashemiana* isolates were genetically characterized in this study.

### 2.2. Molecular Identification

PCR analysis with the species-specific primer pairs TEF-Sb1 and TEF-Sb3 confirmed all 88 isolates as *S. banihashemiana*, as evidenced by the presence of a single specific amplicon of both 443 bp and 577 bp (Table 1, Figure S3). These results were consistent across both the isolates from the culture collection and the newly sampled isolates.

### 2.3. Analysis of Genetic Similarity

A total of 148 DNA fragments were amplified using eight ISSR primers. The characteristics of these primers are outlined in Table 2. From these amplifications, 114 distinct and scorable bands were observed, with fragment sizes ranging from 101 bp to 3150 bp. The M1 primer produced 25 fragments in common, the highest number of bands observed in the experiment. The M2 primer showed the highest percentage of polymorphic loci (*PPL*) at 48.44%. Both the M1 and PCMS primers yielded the highest Shannon index (*I*) values, ranging from 0.548 to 0.587.

**Figure 2:**
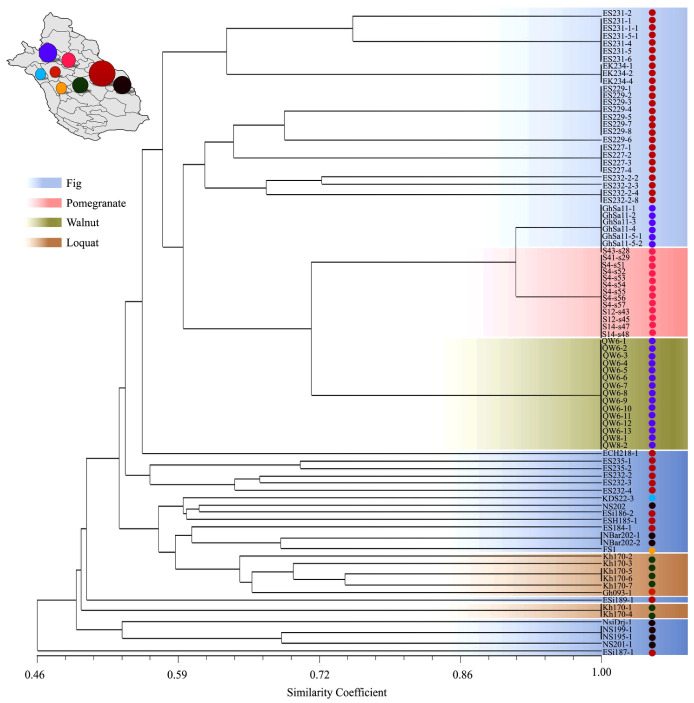
Dendrogram generated by UPGMA-based cluster analysis of ISSR markers of the 88 *Stilbocrea banihashemiana* isolates from infected fig, loquat, pomegranate, and walnut trees. The color-coded map on the upper-left side indicates the geographic distribution of isolates in eight distinct areas of Fars Province. Colored circles indicate sampling locations: blue (Bajgah), red (Estahban), green (Firuzabad), yellow (Kazerun), orange (Khafr), purple (Neyriz), pink (Qalat), and black (Shiraz). Circle size is proportional to the number of isolates obtained per location.

**Figure 3:**
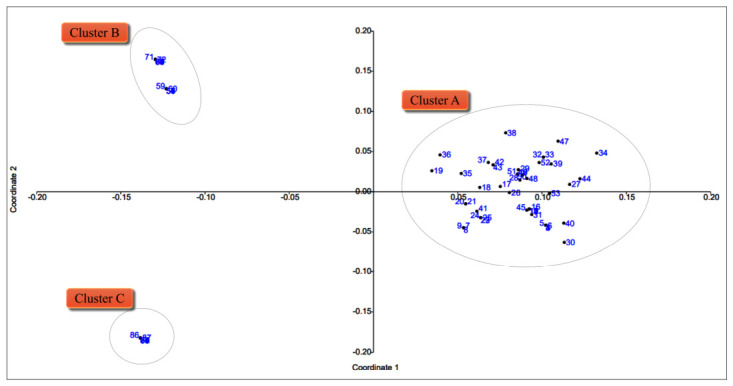
The results of the principal coordinate analysis (PCoA) in the grouping of 88 *Stilbocrea banihashemiana* isolates from infected fig, loquat, pomegranate, and walnut trees in southern Iran, based on eight ISSR markers. Cluster A: 1. ES231-2, 2. ES231-1, 3. ES231-1-1, 4. ES231-5-1, 5. ES231-4, 6. ES231-5, 7. ES231-6, 8. EK234-1, 9. EK234-2, 10. EK234-4, 11. ES229-1, 12. ES229-2, 13. ES229-3, 14. ES229-4, 15. ES229-5, 16. ES229-7, 17. ES229-8, 18. ES229-6, 19. ES232-2-2, 20. ES232-2-3, 21. ES232-2-4, 22. ES232-2-8, 23. ES227-1, 24. ES227-2, 25. ES227-3, 26. ES227-4, 27. ES235-1, 28. ES235-2, 29. ES232-2, 30. ES232-3, 31. ES232-4, 32. NSDrj-1, 33. NS199-1, 34. NS195-1, 35. NS201-1, 36. ESi189-1, 37. ECH218-1, 38. ESi187-1, 39. KDS22-3, 40. NS202, 41. ES184-1, 42. ESi186-1, 43. NBar202-1, 44. NBar202-2, 45. ESH185-1, 46. FS1, 47. Kh170-1, 48. Kh170-2, 49. Kh170-3, 50. Kh170-4, 51. Kh170-5, 52. Kh170-6, 53. Kh170-7, and 54. Gh093-1. Cluster B: 55. GhSa11-1, 56. GhSa11-2, 57. GhSa11-3, 58. GhSa11-4, 59. GhSa11-5-1, 60. GhSa11-5-2, 61. S43-s28, 62. S41-s29, 63. S4-s51, 64. S4-s52, 65. S4-s53, 66. S4-s54, 67. S4-s55, 68. S4-s56, 69. S4-s57, 70. S12-s43, 71. S12-s45, 72. S14-s47, and 73. S14-s48. Cluster C: 74. QW6-1, 75. QW6-2, 76. QW6-3, 77. QW6-4, 78. QW6-5, 79. QW6-6, 80. QW6-7, 81. QW6-8, 82. QW6-9, 83. QW6-10, 84. QW6-11, 85. QW6-12, 86. QW6-13, 87. QW8-1, and 88. QW8-2. Note: The hyphens adjacent to numerical values correspond to minus signs for negative numbers.

Assessing the discriminatory power of ISSR markers revealed that the marker index (*MI*) was lowest for P10 (15.87) and highest for M1 (19.45). The polymorphism information content (*PIC*) ranged from 0.243 (P10) to 0.404 (M1), and the effective multiplex ratio (*EMR*) ranged from 48.65 (P5) to 71.70 (M2). Nei’s Diversity Index (*H*) varied from 0.264 (P10) to 0.402 (M1). Expected heterozygosity (*He*) ranged from 0.246 (P10) to 0.409 (M1), while observed heterozygosity (*H_o_*) ranged from 0.243 (P10) to 0.404 (M1). The effective allele number (*K_e_*) was 1.38 for P10 to 1.70 for M1, and the number of alleles (*A*) was 2 for all primers (Table 3). UPGMA analysis of the ISSR data, at a similarity coefficient of 46%, classified the *S. banihashemiana* isolates into two primary groups. Group I contained only a single isolate, Esi187-1, collected from *F. carica* ‘Siah’ in Estahban County. In contrast, Group II comprised the remaining 87 isolates, which were recovered from infected fig, loquat, pomegranate, and walnut trees across eight locations in Fars Province (Bajgah, Estahban, Firouzabad, Jahrom, Khafr, Neyriz, Qalat, and Shiraz). At the 50% similarity threshold, Group II was further divided into two subgroups. One contained four isolates from fig (‘Sabz’ and ‘Siah’) in Neyriz, while the other encompassed 83 isolates from the various hosts and locations (Figure 2). Principal Coordinate Analysis (PCoA) grouped the 88 isolates into three clusters (Figure 3). Cluster A contained 54 isolates from loquat and various fig cultivars (‘Barg Chenary’, ‘Kalleh Gorbehie’, ‘Puzdonbali’, ‘Sabz’, ‘Shah-Anjeer’, ‘Siah’) collected in the central regions of the province (Estahban, Firuzabad, Kazerun, Khafr, Neyriz, Shiraz). Cluster B included 19 isolates from pomegranate and *F. carica* ‘Sabz’ trees in the northern regions (Bajgah and Qalat). Cluster C consisted of 15 isolates obtained from symptomatic walnut trees in Qalat.

### 2.4. Aggressiveness Assessment of Canker-Causing S. banihashemiana Isolates

**Figure 4:**
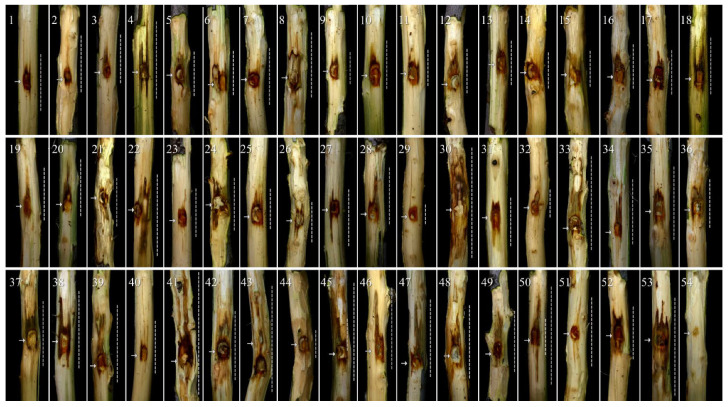
Aggressiveness levels of 53 *Stilbocrea banihashemiana* isolates causing wood necrosis and discoloration on fig detached shoots 14 days after inoculation. Isolates 1–53 correspond to 1. ECH218-1, 2. EK234-1, 3. EK234-2, 4. ES227-1, 5. ES227-2, 6. ES227-3, 7. ES227-4, 8. ES229-1, 9. ES229-2, 10. ES229-3, 11. ES229-4, 12. ES229-5, 13. ES229-6, 14. ES229-8, 15. ES231-1, 16. ES231-1-1, 17. ES231-2, 18. ES231-4, 19. ES231-5, 20. ES231-5-1, 21. ES231-6, 22. ES232-2, 23. ES232-2-2, 24. ES232-2-3, 25. ES232-2-8, 26. ES232-3, 27. ES232-4, 28. ES235-1, 29. ES235-2, 30. Esi186-1, 31. FS1, 32. Gh093-1, 33. GhSa11-1, 34. GhSa11-2, 35. GhSa11-5-1, 36. Kh170-1, 37. Kh170-2, 38. Kh170-4, 39. Kh170-6, 40. Kh170-7, 41. NS195-1, 42. NS199-1, 43. NS202, 44. NSDrj-1, 45. QW6-1, 46. QW6-10, 47. QW8-1, 48. QW8-2, 49. S12-s43, 50. S14-s47, 51. S41-s29, 52. S43-s28, and 53. S4-s51. The negative control was inoculated with non-colonized PDA (54). Dashed lines demarcate the boundaries of necrotic and discolored tissue. Arrows represent the locations of the inoculation sites.

The results of the pathogenicity assay of 53 representative *S. banihashemiana* isolates on detached shoots confirmed that all examined isolates could induce wood necrosis and discoloration on *F. carica* ‘Sabz’ (Figure 4). The ANOVA revealed that *S. banihashemiana* isolates were significantly different from each other (*p*  <  0.0001) in the average four pathogenicity traits, including LL, LW, UILP, and DILP (Table S1).

**Figure 5:**
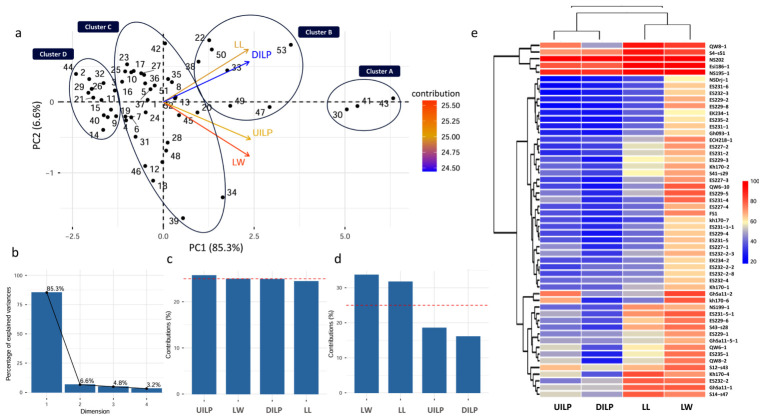
Principal component analysis (PCA) and heatmap of the aggressiveness assessment dataset of *Stilbocrea banihashemiana* isolates on detached fig shoots. (**a**) The PCA biplot, including the first (PC1) and the second (PC2) principal components categorized the isolates into four groups based on the four pathogenicity traits: Cluster A, highly aggressive isolates (30: Esi186-1, 41: NS195-1, and 43: NS202); Cluster B, aggressive isolates (22: ES232-2, 33: GhSa11-1, 38: Kh170-4, 47: QW8-1, 49: S12-s43, 50: S14-s47, and 53: S4-s51); Cluster C, moderately aggressive isolates (1: ECH218-1, 4: ES227-1, 5: ES227-2 6: ES227-3, 7: ES227-4, 8: ES229-1, 10: ES229-3, 12: ES229-5, 13: ES229-6, 16: ES231-1-1, 17: ES231-2, 18: ES231-4, 20: ES231-5-1, 23: ES232-2-2, 24: ES232-2-3, 25: ES232-2-8, 27: ES232-4, 28: ES235-1, 31: FS1, 34: GhSa11-2, 35: GhSa11-5-1, 36: Kh170-1, 37: Kh170-2, 39: kh170-6, 42: NS199-1, 45: QW6-1, 46: QW6-10, 48: QW8-2, 51: S41-s29, and 52: S43-s28); and Cluster D, less aggressive isolates (2: EK234-1, 3: EK234-2, 9: ES229-2, 11: ES229-4, 14: ES229-8, 15: ES231-1, 19: ES231-5, 21: ES231-6, 26: ES232-3, 29: ES235-2, 32: Gh093-1, 40: kh170-7, and 44: NSDrj-1). (Note: the hyphens adjacent to numerical values correspond to minus signs for negative numbers. (**b**) The percentage of explained variance for each of the four dimensions (principal components). (**c**,**d**) The bar chart displays the contribution of each pathogenicity trait, including lesion length (LL), lesion width (LW), upward internal lesion progression (UILP), and downward internal lesion length progression (DILP) to the first (**c**) and second (**d**) principal components. The red dashed line in the graph indicates the expected average contribution. (**e**) The heatmap shows the percentages that represent each pathogenicity trait’s severity, calculated using the formula: [(value of each pathogenicity trait/maximum value in that trait) × 100].

The most aggressive isolates, Esi186-1, NS202, and NS195-1, produced the largest lesion dimensions. In contrast, isolate NSDrj-1 was among the least aggressive, exhibiting the smallest four pathogenicity traits (Table S2). Principal component analysis (PCA) of the aggressiveness data classified the 53 *S.* *banihashemiana* isolates into four distinct clusters based on four pathogenicity traits (Figure 5). The first two principal components (PC1 and PC2) explained 91.9% of the total variance (85.3% and 6.6%, respectively) (Figure 5b). The primary drivers of variation were UILP and LW, loading most heavily on PC1 and PC2, respectively (Figure 5c,d). Furthermore, the PCA biplot revealed a strong positive correlation between UILP and LL, as well as between DILP and LW, as indicated by the acute angle between their vectors (Figure 5a). The clustering aligned closely with the isolates’ levels of aggressiveness: Cluster A, highly aggressive isolates (Esi186-1, NS195-1, and NS202); Cluster B, aggressive isolates (ES232-2, GhSa11-1, Kh170-4, QW8-1, S12-s43, S14-s47, and S4-s51); Cluster C, moderately aggressive isolates (ECH218-1, ES227-1, 5: ES227-2, ES227-3, ES227-4, ES229-1, ES229-3, ES229-5, ES229-6, ES231-1-1, ES231-2, ES231-4, ES231-5-1, ES232-2-2, ES232-2-3, ES232-2-8, ES232-4, ES235-1, FS1, GhSa11-2, GhSa11-5-1, Kh170-1, Kh170-2, Kh170-6, NS199-1, QW6-1, QW6-10, QW8-2, S41-s29, and S43-s28); and less aggressive isolates (EK234-1, EK234-2, ES229-2, ES229-4, ES229-8, ES231-1, ES231-5, ES231-6, ES232-3, ES235-2, Gh093-1, Kh170-7, and NSDrj-1). This clustering pattern was further corroborated by a heatmap generated from the four pathogenicity traits (Figure 5e).

### 2.5. Susceptibility of Fig Cultivars to S. banihashemiana

**Figure 6:**
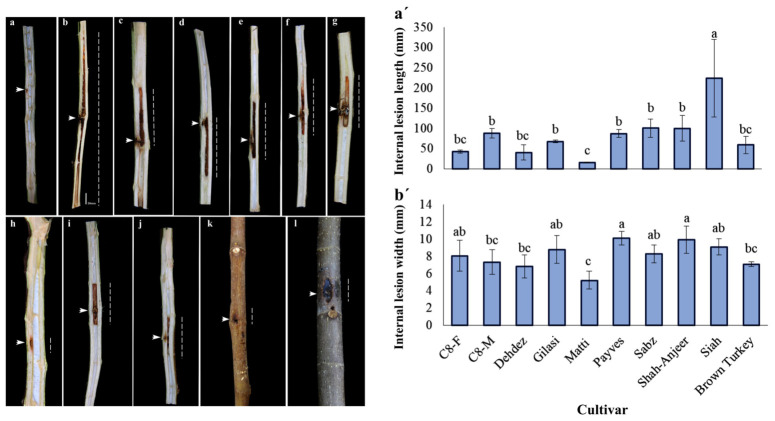
Variation in susceptibility among fig cultivars six months after inoculation with *Stilbocrea banihashemiana* isolate FS1 (ex-type isolate; CBS 148864). Susceptibility was evaluated based on the internal length and width of wood discoloration. Panels show: (**a**) negative control (*Ficus carica* ‘Sabz’); (**b**–**j**) Internal wood discoloration in nine inoculated cultivars: ‘Siah’, ‘Shah-Anjeer’, ‘Sabz’, ‘Payves’, ‘Gilasi’, ‘Brown Turkey’, ‘Matti’, ‘C8-F’, and ‘Dehdez’. (**k**,**l**) External wood discoloration symptoms on the bark of cultivars ‘Siah’, and ‘Sabz’, respectively. White arrows indicate the inoculation sites, and white dashed lines indicate the longitudinal internal progression of wood discoloration. The distribution of mean internal lesion length (**a’**) and internal lesion width (**b’**) was measured among the 10 inoculated cultivars. Bars represent mean values, and error bars indicate the standard deviation of three replicates. Different letters above the bars denote statistically significant differences among cultivars at *p* ≤ 0.05.

Significant differences in susceptibility to *S. banihashemiana* were observed among the evaluated fig cultivars following artificial wound-inoculation (Figure 6). One-way analysis of variance (ANOVA) revealed that cultivar had a significant effect on both pathogenicity parameters: internal lesion length and width, three months post-inoculation (*p* < 0.05; Table S3). Tukey’s honestly significant difference (HSD) test grouped the fig cultivars into distinct statistical subsets based on lesion development, revealing clear variation in disease severity. Internal lesion length differed markedly among cultivars; ‘Siah’ exhibited the highest susceptibility, with a mean lesion length of 224.7 mm, whereas ‘Matti’ showed the lowest lesion length (15.9 mm), indicating comparatively minimal susceptibility (Figure 6a’,b’). Internal lesion width showed a similar trend, although the relative ranking of cultivars differed slightly. The greatest internal lesion width was observed in ‘Shah-Anjeer’ (10.0 mm), whereas the narrowest lesions were recorded in ‘Matti’ (5.24 mm) three months post-inoculation (Figure 6a’,b’).

## 3. Discussion

This study is a comprehensive investigation of multiple biological and epidemiological aspects of *S. banihashemiana*, an emerging canker and dieback pathogen affecting economically important fruit trees in Iran. In this research, we combined molecular and morphological identification of isolates from multiple natural hosts, genetic analysis at population-level across diverse hosts and regions, evaluation of isolate aggressiveness, and assessment of fig cultivar susceptibility. Together, these approaches provided a better insight into the pathogen biology and ecology. Critical to this study was the unambiguous confirmation of species identity for all isolates prior to detailed population genetic analysis. The use of *S. banihashemiana*-specific primers (TEF-Sb1 and TEF-Sb3) consistently amplified the expected fragments across the isolates, confirming they represent a single phylogenetic species. This preliminary verification step, often overlooked in population studies, ensured that the genetic diversity and aggressiveness patterns observed reflect true intraspecific variation rather than cryptic species admixture, a particularly important consideration given the recent description of this species [1] and the morphological plasticity within *Bionectriaceae* [32].

The aggressiveness assessment of 53 representative isolates on detached fig shoots revealed highly significant variation across all four pathogenicity traits measured. This quantitative variation, ranging from highly aggressive to less aggressive isolates, demonstrates that *S. banihashemiana* populations harbor substantial pathogenic potential that could respond to selection pressure from management practices or host resistance. Notably, isolates from northern Fars (Bajgah and Qalat) were disproportionately represented in the highly aggressive and aggressive clusters, despite their genetic distinctiveness in PCoA. This geographic concentration of high aggressiveness may indicate either local adaptation to environmental conditions or the presence of particularly virulent genotypes in this region. Comparable patterns of intra-population aggressiveness variability of *S. banihashemiana* were reported by Sekandarpour et al. [3] in pomegranate, where isolates were clearly separated into highly, moderately, and less-aggressive clusters based on upward lesion progression (ULP), downward lesion progression (DLP), and lesion width. From a management perspective, the identification of highly aggressive isolates provides critical material for resistance screening programs and for studying the molecular basis of pathogenicity. The detached shoot assay proved to be a reliable and efficient method for aggressiveness screening, consistent with previous applications for other canker pathogens, such as *Diaporthe cinerascens* [14], *Neonectria ditissima* (Tul. & C. Tul.) Samuels & Rossman [33], and *Botryosphaeriaceae* species on pistachio [34].

The highly significant differences in susceptibility to *S. banihashemiana* observed among the 10 fig cultivars demonstrate substantial genetic variability in response to this fungal pathogen. Internal lesion length and width, measured three months after inoculation with the ex-type isolate, proved to be reliable indicators of host susceptibility, particularly given the importance of subcortical pathogen progression in canker diseases. Cultivars were clearly separated into three susceptibility groups. ‘Siah’ exhibited high susceptibility, with rapid internal colonization (>220 mm lesion length), whereas ‘Matti’ showed minimal lesion development (<16 mm), indicating strong restriction of pathogen progression. Most commercial cultivars, including the dominant dried fig ‘Sabz’, displayed moderate susceptibility in line with previous studies [14,24]. Comparable patterns of cultivar differentiation against *S. banihashemiana* were reported by Sekandarpour et al. [3] in pomegranate, where susceptibility was assessed using six pathogenicity traits, including internal lesion length and vascular progression. In that study, internal vascular colonization traits were the primary contributors to phenotypic variance in PCA, reinforcing the central role of systemic wood invasion, rather than superficial lesion size alone, as an indicator of host susceptibility. The consistent least susceptibility of ‘Matti’ across multiple canker pathogens reported in previous studies [14,24] suggests the presence of broad-spectrum resistance mechanisms. These mechanisms may involve constitutive structural barriers such as enhanced lignification and periderm formation [35], as well as inducible biochemical defenses including phenolic accumulation via the phenylpropanoid pathway [36], and activation of pathogenesis-related (PR) proteins [37,38]. In contrast, the high susceptibility of ‘Siah’, despite reported abiotic stress tolerance, confirms that canker resistance is not necessarily linked to abiotic stress adaptation, highlighting the need for direct pathological screening. From a practical perspective, moderate susceptibility in commercial cultivars may be manageable under integrated disease management strategies; however, highly susceptible genotypes pose a significant risk in areas with high inoculum pressure. From a breeding standpoint, the strong phenotypic contrast between ‘Matti’ and ‘Siah’ provides valuable material for genetic studies and resistance improvement programs [39]. Recent molecular approaches in fruit crop–pathogen systems demonstrate that integrating genome-wide association studies (GWAS) with transcriptomic analyses significantly accelerates the identification of resistance markers and candidate genes [40,41]. Although the species and pathogens examined in previous studies differ from those in the present context, these advances establish a framework for future research. Specifically, research should progress beyond conventional pathological phenotypic assessments, such as measuring lesion size, lignification, and phenolic content, toward GWAS and transcriptomic profiling of contrasting cultivars, including ‘Matti’ and ‘Siah’. These strategies have the potential to identify quantitative trait loci and candidate genes, thereby facilitating marker-assisted or genomic selection for canker resistance in fig breeding programs. Integrating stable, less susceptible germplasm with advanced molecular tools constitutes an essential step toward achieving durable resistance in fig trees.

The genetic diversity analysis using eight ISSR markers revealed significant genotypic variation among the 88 *S. banihashemiana* isolates, with similarity coefficients ranging from 0.46 to 1.00. The pathogen’s ecological diversity is well represented by its broad host range, including fig, loquat, pomegranate, and walnut, and its wide geographic distribution across eight regions (Bajgah, Estahban, Firuzabad, Kazerun, Khafr, Neyriz, Qalat, and Shiraz). Furthermore, the dataset breadth and representativeness are enhanced by the inclusion of isolates from various fig cultivars, such as the dried fig cultivar ‘Sabz’, the fresh-fruit cultivars ‘Barg Chenary’, ‘Kalleh Gorbehie’, ‘Shah-Anjeer’, and ‘Siah’, as well as the caprifig ‘Puzdonbali’. This wide range of genetic distances suggests the presence of multiple divergent genotypes within the population rather than a clonal lineage structure. Although sexual reproduction was not observed during field surveys, the high genetic diversity detected in *S. banihashemiana* suggests that additional sources of variation may be present. Mechanisms such as parasexual recombination, aneuploidy, transposable-element activity, stress-associated mutagenesis, and epigenetic regulation are known to generate diversity in other asexual fungi [42,43,44,45,46,47]. However, these mechanisms were not evaluated in this study and should be considered as hypotheses for future investigation rather than established processes. This observed diversity pattern contrasts with the low-variability, predominantly clonal structure previously reported for fig Diaporthe canker [22].

The absence of host-specific clustering in the UPGMA dendrogram, where isolates from fig, pomegranate, loquat, and walnut were intermixed across clusters, provides compelling evidence that *S. banihashemiana* behaves as a host-generalist pathogen rather than exhibiting strict host specialization. This pattern aligns with traits observed in other *Bionectriaceae* members, including *S. walteri* from multiple *Citrus* spp. [8] and *S. macrostoma* from various woody hosts [9], suggesting that generalist life strategies may be evolutionarily conserved within this genus.

The PCoA analysis further divided the population into three major clusters that partially corresponded to geographic origin and host species. Cluster A contained 61% of all isolates, mainly from fig cultivars and loquat collected across six central and southern districts of Fars Province. This pattern suggests that these areas may serve as important regional centers of population aggregation or genetic diversity; however, ISSR-based clustering alone cannot substantiate inferences about ancestral ranges or primary centers of evolutionary origin. Such interpretations require dedicated phylogeographic analyses, including molecular dating or ancestral area reconstruction. The relatively high genetic similarity among isolates within Cluster A (approximately 70%) suggests substantial gene flow across these districts, potentially facilitated by long-distance dispersal mechanisms such as movement of infected nursery stock, contaminated pruning tools, or airborne conidia. In contrast, isolates obtained from pomegranate, walnut, and fig trees (specifically the ‘Sabz’ cultivar) in northern districts formed two distinct clusters, consistent with the UPGMA dendrogram. Pomegranate and ‘Sabz’ fig isolates grouped in Cluster B and exhibited approximately 92% similarity, whereas walnut isolates consistently formed a separate group in both analyses.

The broad host range of *S. banihashemiana*, as documented by Negahban et al. [48], suggests that the observed genotypic variation within this pathogen’s populations across diverse hosts may result from the pathogen’s successful colonization of new hosts and subsequent environmental adaptation [49]. In contrast, the genetic homogeneity detected within *D. cinerascens* populations, the causal agent of fig Diaporthe canker, is likely due to the pathogen’s host specificity to fig trees [14,17,21]. Additionally, the varying degrees of aggressiveness exhibited by isolates from different hosts and fig cultivars, on detached branches of their natural hosts [48], along with the ability to induce a range of symptoms from types of C & D cankers sensu Bolboli et al. [14] to dieback and internal discoloration of wood [4], may be intricately linked to the genotypic diversity of *S. banihashemiana*.

## 4. Materials and Methods

### 4.1. Preparation of Fungal Isolates

A total of 88 isolates of *S. banihashemiana* were used in this study: 51 of them were from the culture collection of the Department of Plant Protection at Shiraz University and were previously recovered from various fruit trees exhibiting canker and dieback symptoms, including fig and loquat [1], pomegranate [3], and walnut [4] in southern Iran (Table 1), while additional isolates were sampled in this study, in February and August 2023, from commercial fig plantations in the central districts of Fars Province. Samples were collected from trees showing symptoms of Bionectria canker (type C, and D sensu [14]) and dieback. Symptomatic tissues were transported to the laboratory for fungal isolation. Following stem dissection, wood pieces were excised from the margin between necrotic and healthy tissues for culturing, as described by Tavakolian et al. [50]. The research encompassed isolates from eight distinct geographical locations: Bajgah, Estahban, Firuzabad, Kazerun, Khafr, Neyriz, Qalat, and Shiraz (Figure 1, Table 1). Fig-derived isolates originated from several cultivars: ‘Sabz’ (predominantly used for drying), the fresh fig cultivars ‘Barg Chenary’, ‘Kalleh Gorbehie’, ‘Shah-Anjeer’, and ‘Siah’, as well as the caprifig ‘Puzdonbali’ [1] (Table 1).

### 4.2. DNA Extraction and PCR-Based Identification

Prior to DNA extraction, all isolates were cultured in potato broth (PB; extract of 300 g of potatoes in one liter of distilled water) for 20 days at room temperature. The resulting mycelium was harvested, freeze-dried, and ground into a fine powder. Total genomic DNA was then extracted using the DNG-PLUS extraction kit (CinnaGen, Tehran, Iran) following the protocol provided by Mirsoleimani and Mostowfizadeh-Ghalamfarsa [51]. The concentration and purity of the extracted DNA were determined using an MD-1000 Nanodrop spectrophotometer (NanoDrop Technologies, Wilmington, DE, USA). All isolates were confirmed to be *S. banihashemiana* through PCR-based assay using the species-specific primer pairs TEF-Sb1 and TEF-Sb3 (Table S4), which target the *tef1* gene [52]. PCR reactions were carried out in a final volume of 25 µL containing 100 ng of genomic DNA (1 µL), 10 pmol of each primer, 12.5 µL of 2× Taq DNA Polymerase Master Mix RED (Ampliqon, Odense, Denmark), and 9.5 µL of PCR-grade water. PCR amplification was performed in a Peltier Thermal Cycler (Bio-Techne, Minneapolis, MN, USA) under the following conditions: an initial denaturation at 95 °C for 2 min; 35 cycles of denaturation at 95 °C for 60 s, annealing at 65 °C for 60 s, and extension at 72 °C for 50 s; and a final extension at 72 °C for 10 min [1,52]. PCR products were separated by electrophoresis on a 1% (*w*/*v*) agarose gel stained with 0.05% ethidium bromide. A 100 bp DNA ladder (GeneRuler, Fermentas, Vilnius, Lithuania) was used as a molecular size standard. The DNA bands were visualized and documented under ultraviolet light using a Syngene gel documentation system (Syngene, Frederick, MD, USA).

### 4.3. Analysis of the Inter-Simple Sequence Repeats (ISSR)-PCR

Following PCR-based identification of the isolates, genetic diversity among *S. banihashemiana* isolates was assessed using eight ISSR primers [26,27,28,29,30,31] (Table 2). The ISSR-PCR amplification protocol consisted of an initial denaturation step at 95 °C for 5 min, followed by 35 amplification cycles of denaturation at 95 °C for 55 s, primer-specific annealing for 90 s, and extension at 72 °C for 50 s. A final extension step was performed at 72 °C for 10 min. PCR reactions were carried out in a total volume of 25 µL, as described in Section 4.2. The molecular weights of amplified fragments were estimated by comparing band sizes with a molecular marker using GelAnalyzer 19.1 software (Istvan Lazar, Hungary). Only clear and well-resolved bands were considered for further analysis. Polymorphism was evaluated by constructing a binary data matrix, in which the presence or absence of bands was scored as 1 or 0, respectively. A similarity matrix was generated and subjected to cluster analysis using the unweighted pair group method with arithmetic averages (UPGMA). Corresponding dendrograms were constructed using NTSYS-pc version 2.1 software [53].

The discriminatory capacity of each ISSR primer was evaluated using 10 statistical parameters. The percentage of polymorphic loci (*PPL*) and the number of alleles (*A*) were calculated for each primer. Polymorphic information content (*PIC*) was determined using the formula *PIC*_i_ = 2f_i_ (1 − f_i_), where *PIC*_i_ represents the polymorphic information content of marker *i*, *f_i_* is the frequency of band presence, and (1 − *f_i_*) is the frequency of band absence [54]. The marker index (*MI*), which reflects the overall efficiency of the marker system, was calculated as *MI* = *EMR* × *PIC* [55]. The effective multiplex ratio (*EMR*) was computed as *EMR* = *n* × *β*, where *n* is the average number of fragments amplified per accession and *β* is defined as *PB*/(*PB* + *MB*), with *PB* representing polymorphic bands and *MB* representing monomorphic bands. Alternatively, *EMR* was calculated following Powell et al. [56] as *EMR* = *n_p_* (*n_p_*/*n*), where *n_p_* is the number of polymorphic fragments. Genetic diversity indices, including expected heterozygosity (*H_e_*), observed heterozygosity (*H_o_*), Nei’s genetic diversity (*H*), effective number of alleles (*K_e_*), and Shannon’s information index (*I*), were calculated using Popgene version 1.32 software [57]. In addition, principal coordinate analysis (PCoA) was performed using DARwin 5.0 software to visualize the genetic relationships among the isolates in a scatter plot [58].

### 4.4. Pathogenicity Assay of S. banihashemiana Isolates

Among the 88 total isolates (Table 1), 53 were selected for the pathogenicity assay (Table 2). The goal was to include all four host species (fig, loquat, pomegranate, and walnut) and all eight geographical locations while avoiding pseudo-replication from identical GPS/date combinations. For fig (the primary host, overall 52 isolates), 38 were retained to represent all recorded cultivars (‘Sabz’, ‘Kalleh Gorbehie’, ‘Shah-Anjeer’, ‘Barg Chenary’, ‘Siah’, and ‘Puzdonbali’) and all five geographical locations. For the less abundant non-fig hosts (overall 36 isolates), a smaller subset was chosen to provide preliminary host-range comparisons: loquat 6 of 8 isolates, pomegranate 5 of 13, and walnut 4 of 15. This design ensures that every host species and location is represented (Table S2). Pathogenicity tests were carried out using detached fig tree shoots inoculated with the selected isolates. Dormant shoots (approximately 10 mm in diameter) were gathered from fig trees of the ‘Sabz’ cultivar, the most commonly grown commercial dried fig cultivar in the region [13]. The shoots were cut into 25–30 cm segments and surface-disinfected with 98% industrial ethanol followed by flaming. At each inoculation site, the bark was removed using a sterile cork borer (6 mm diameter). A mycelial plug (5 × 5 × 2 mm) taken from the actively growing edge of a 7-day-old culture on potato dextrose agar (PDA; 300 g/L boiled potato extract, 20 g/L glucose monohydrate, 15 g/L agar, distilled water) was inserted into the wound. The original bark disk was replaced, the inoculation site was sealed with Parafilm^®^ (Bemis Packaging, Sheboygan Falls, WI, USA) to prevent drying out and contamination, and one end of the shoot was sealed with paraffin wax to minimize water loss. Control treatments included sterile, non-colonized PDA plugs applied in the same procedure. Five detached shoots were used for each isolate, as replicates, arranged in a completely randomized design (CRD). Inoculated and control shoots were placed individually in glass bottles with 100 mL of sterile distilled water and kept in a growth chamber at 25 ± 2 °C under a 16 h light/8 h dark photoperiod.

Disease evaluations were conducted 14 days after inoculation. External symptoms were recorded, and then the bark was removed carefully to assess internal wood discoloration. Lesion dimensions were measured using a digital caliper (Insize^®^, Suzhou, China, with 0.01 mm precision). To fulfill Koch’s postulates, five wood pieces (5 × 5 mm) were excised from the margin of each necrotic lesion, surface-sterilized, and plated onto PDA amended with tetracycline [14]. Isolate aggressiveness was quantified based on four key pathogenicity parameters: lesion length (LL), lesion width (LW), upward internal lesion progression (UILP), and downward internal lesion progression (DILP), all measured in millimeters. These traits have been recognized as reliable indicators of pathogenic aggressiveness in previous research [14,33,34,59].

Prior to conducting statistical analysis, data normality for each pathogenicity trait was assessed using the Shapiro–Wilk test [60] in IBM SPSS Statistics version 25. The effect of fungal isolates on each of the four pathogenicity traits was evaluated using one-way analysis of variance (ANOVA) within the General Linear Model (GLM) framework in SAS version 9.4 (SAS Institute, Cary, NC, USA). When significant differences were detected (*p* ≤ 0.05), mean comparisons were performed using Tukey’s Honestly Significant Difference (HSD) post hoc test. To explore relationships among the four pathogenicity traits and to visualize the relative aggressiveness of the isolates, multivariate analyses were conducted. Principal component analysis (PCA) was performed on the dataset comprising 53 *S. banihashemiana* isolates using R software version 3.4., with the “FactoMineR” [61] and “factoextra” [62] packages. Additionally, a heatmap was generated using the “pheatmap” package [63] to provide an integrated visualization of disease severity across all isolates and traits. For this purpose, raw data for each trait were normalized to a 0–100 scale by dividing each value by the maximum observed value for that trait and multiplying by 100, enabling direct comparison of relative severity among traits.

### 4.5. Susceptibility Assessment of Fig Cultivars to S. banihashemiana

The susceptibility of fig cultivars to *S. banihashemiana* was evaluated using one-year-old saplings of 10 cultivars selected based on their economic importance and reported tolerance to drought and salinity. The tested cultivars included commercial dried fig cultivars (‘Sabz’ and ‘Payves’), fresh fig cultivars (‘Matti’, ‘Shah-Anjeer’, ‘Siah’, and ‘Brown Turkey’), wild cultivars (‘Dehdez’ and ‘Gilasi’), and hybrid cultivars (‘C8-M’ [‘Sabz’ × ‘Khormaei’] and ‘C8-F’ [‘Sabz’ × ‘Khormaei’]) [14,64,65,66,67,68]. Herbarium voucher specimens at the Seed and Plant Certification and Registration Institute, Iran, are under the following accession numbers: I.R.IRAN 3.4.1.11 (*F. carica* ‘Matti’), I.R.IRAN 3.4.1.3 (F. carica ‘Payves’), I.R.IRAN 3.4.1.6 (*F. carica* ‘Sabz’), I.R.IRAN 3.4.1.7 (*F. carica* ‘Siah’), I.R.IRAN 3.4.1.9 (*F. carica* ‘Shah-Anjeer’), and I.R.IRAN 3.4.1.8 (*F. carica* ‘Brown Turkey’).

Pathogenicity assays were conducted using the ex-type isolate of *S. banihashemiana* (FS1 = CBS 148864). The isolate was grown on PDA and incubated at 25 ± 2 °C for seven days. Conidia were harvested by transferring a plug of actively sporulating culture into a 1.5 mL microcentrifuge tube, adding 1 mL of sterile distilled water, and agitating the suspension thoroughly. The suspension was filtered through a double layer of sterile cheesecloth, and conidial concentration was determined using a hemocytometer and adjusted to 1 × 10^6^ conidia/mL with sterile distilled water [69]. Artificial inoculation was performed by drilling wounds (2 mm diameter × 3 mm depth) into the stem approximately 1 cm below the apical node using a power drill with a sterilized bit. Each wound received 20 µL of the conidial suspension and was immediately sealed with a mixture of Vaseline (Unilever Company, Netherlands) and Parafilm. Control plants were mock-inoculated with 20 µL of sterile distilled water [13]. Each cultivar was represented by three biological replicates. Following inoculation, saplings were maintained under controlled greenhouse conditions and monitored daily for symptom development over three months.

At three months post-inoculation, pathogenicity was quantified by measuring internal lesion length (ILL) and width (ILW) beneath the stem bark, which are reliable indicators of disease severity in fig canker [13,45]. The measurements were taken with a digital caliper after carefully removing the bark to expose necrotic tissues. Cultivar susceptibility was determined by comparing lesion dimensions among the inoculated plants. To confirm the identity of the pathogen and fulfill Koch’s postulates, *S. banihashemiana* was re-isolated from symptomatic stem tissues on PDA supplemented with tetracycline. The recovered isolates were identified based on their cultural and morphological characteristics and compared to the original inoculum [1].

The experiment was conducted using a completely randomized design with three replications. Data normality for lesion length and width was evaluated using the Shapiro–Wilk test (see Section 4.4). Since there was deviation from normality, lesion length data was square-root transformed prior to analysis. The effects of cultivar on lesion length and width were analyzed using one-way analysis of variance (ANOVA), followed by Tukey’s honestly significant difference (HSD) test, at *p* < 0.05. All statistical analyses were carried out using SAS software (version 9.4). Graphical representations were generated using Microsoft Excel.

## 5. Conclusions

This study demonstrates that *S. banihashemiana* populations harbor substantial genetic and phenotypic diversity across hosts and regions, despite the absence of observed sexual reproduction. High genotypic variation, coupled with variable aggressiveness among isolates and broad host generalism, indicates significant evolutionary potential and adaptive flexibility. While some mechanisms are possible contributors to this diversity, further genomic and cytological studies are needed to confirm their roles. The identification of ‘Matti’ as a less-susceptibility cultivar provides an immediate resource for resistance fig breeding programs. Given its consistent performance across multiple canker pathogens, ‘Matti’ could serve as a donor parent in hybridization efforts to improve the disease resistance of commercially desirable but susceptible cultivars, such as ‘Sabz’. Collectively, these findings establish a foundational framework for predicting disease dynamics, informing management strategies, and guiding future epidemiological, evolutionary, and functional studies of *S. banihashemiana*.

## Figures and Tables

**Table 1:** List of *Stilbocrea banihashemiana* isolates used in this study, recovered from infected fruit trees in eight different locations of Fars Province, Iran.

Isolate	Location	Host	Date	Latitude	Longitude	Reference
ES231-1	Estahban	*Ficus carica* ‘Sabz’	7-February-2023	29.09478	54.05592	This study
ES231-2	Estahban	*F. carica* ‘Sabz’	7-February-2023	29.09478	54.05592	This study
ES231-1-1	Estahban	*F. carica* ‘Sabz’	7-February-2023	29.09478	54.05592	This study
ES231-5-1	Estahban	*F. carica* ‘Sabz’	7-February-2023	29.09478	54.05592	This study
ES231-4	Estahban	*F. carica* ‘Sabz’	7-February-2023	29.09478	54.05592	This study
ES231-5	Estahban	*F. carica* ‘Sabz’	7-February-2023	29.09478	54.05592	This study
ES231-6	Estahban	*F. carica* ‘Sabz’	7-February-2023	29.09478	54.05592	This study
EK234-1	Estahban	*F. carica* ‘Kalleh Gorbehie’	7-February-2023	29.06885	54.04481	This study
EK234-2	Estahban	*F. carica* ‘Kalleh Gorbehie’	7-February-2023	29.06885	54.04481	This study
EK234-4	Estahban	*F. carica* ‘Kalleh Gorbehie’	7-February-2023	29.06885	54.04481	This study
ES229-1	Estahban	*F. carica* ‘Sabz’	7-February-2023	29.09471	54.05609	This study
ES229-2	Estahban	*F. carica* ‘Sabz’	7-February-2023	29.09471	54.05609	This study
ES229-3	Estahban	*F. carica* ‘Sabz’	7-February-2023	29.09471	54.05609	This study
ES229-4	Estahban	*F. carica* ‘Sabz’	7-February-2023	29.09471	54.05609	This study
ES229-5	Estahban	*F. carica* ‘Sabz’	7-February-2023	29.09471	54.05609	This study
ES229-6	Estahban	*F. carica* ‘Sabz’	7-February-2023	29.09471	54.05609	This study
ES229-7	Estahban	*F. carica* ‘Sabz’	7-February-2023	29.09471	54.05609	This study
ES229-8	Estahban	*F. carica* ‘Sabz’	7-February-2023	29.09471	54.05609	This study
ES232-2-2	Estahban	*F. carica* ‘Sabz’	7-February-2023	29.09495	54.05591	This study
ES232-2-3	Estahban	*F. carica* ‘Sabz’	7-February-2023	29.09495	54.05591	This study
ES232-2-4	Estahban	*F. carica* ‘Sabz’	7-February-2023	29.09495	54.05591	This study
ES232-2-8	Estahban	*F. carica* ‘Sabz’	7-February-2023	29.09495	54.05591	This study
ES227-1	Estahban	*F. carica* ‘Sabz’	7-February-2023	29.09482	54.05622	This study
ES227-2	Estahban	*F. carica* ‘Sabz’	7-February-2023	29.09482	54.05622	This study
ES227-3	Estahban	*F. carica* ‘Sabz’	7-February-2023	29.09482	54.05622	This study
ES227-4	Estahban	*F. carica* ‘Sabz’	7-February-2023	29.09482	54.05622	This study
ES235-1	Estahban	*F. carica* ‘Shah-Anjeer’	7-February-2023	29.06885	54.04481	This study
ES235-2	Estahban	*F. carica* ‘Shah-Anjeer’	7-February-2023	29.06885	54.04481	This study
ES232-2	Estahban	*F. carica* ‘Sabz’	7-February-2023	29.06885	54.05591	This study
ES232-3	Estahban	*F. carica* ‘Sabz’	7-February-2023	29.06885	54.05591	This study
ES232-4	Estahban	*F. carica* ‘Sabz’	7-February-2023	29.06885	54.05591	This study
GhSa11-1	Qalat	*F. carica* ‘Sabz’	21-August-2023	29.81839	52.32295	This study
GhSa11-2	Qalat	*F. carica* ‘Sabz’	21-August-2023	29.81839	52.32295	This study
GhSa11-3	Qalat	*F. carica* ‘Sabz’	21-August-2023	29.81832	52.32293	This study
GhSa11-4	Qalat	*F. carica* ‘Sabz’	21-August-2023	29.81832	52.32293	This study
GhSa11-5-1	Qalat	*F. carica* ‘Sabz’	21-August-2023	29.81832	52.32293	This study
GhSa11-5-2	Qalat	*F. carica* ‘Sabz’	21-August-2023	29.81838	52.32294	This study
NSDrj-1	Neyriz	*F. carica* ‘Sabz’	18-November-2020	29.08777	54.17480	[1]
NS199-1	Neyriz	*F. carica* ‘Sabz’	23-October-2019	29.08755	54.17512	[1]
NS195-1	Neyriz	*F. carica* ‘Sabz’	23-October-2019	29.08077	54.17512	[1]
NS201-1	Neyriz	*F. carica* ‘Sabz’	23-October-2019	29.08730	54.17512	[1]
ESi189-1	Estahban	*F. carica* ‘Sabz’	23-October-2019	29.06856	54.04583	[1]
ECH218-1	Estahban	*F. carica* ‘Barg Chenary’	18-November-2020	29.06858	54.04549	[1]
ESi187-1	Estahban	*F. carica* ‘Siah’	23-October-2019	29.06873	54.04584	[1]
KDS22-3	Kazerun	*F. carica* ‘Sabz’	8-January-2019	29.49482	51.47688	[1]
NS202	Neyriz	*F. carica* ‘Sabz’	23-October-2019	29.08759	54.17470	[1]
ES184-1	Estahban	*F. carica* ‘Sabz’	23-October-2019	29.06794	54.04533	[1]
ESi186-1	Estahban	*F. carica* ‘Siah’	23-October-2019	29.06873	54.04563	[1]
NBar202-1	Neyriz	*F. carica* ‘Puzdonbali’	23-October-2019	29.08777	54.17480	[1]
NBar202-2	Neyriz	*F. carica* ‘Puzdonbali’	23-October-2019	29.08777	54.17480	[1]
ESH185-1	Estahban	*F. carica* ‘Shah-Anjeer’	23-October-2019	29.06852	54.04596	[1]
FS1 *	Firuzabad	*F. carica* ‘Sabz’	18-November-2020	28.49198	52.33396	[1]
Kh170-1	Khafr	*Eryobotria japonica*	1-October-2019	28.59053	53.12299	[1]
Kh170-2	Khafr	*E. japonica*	1-October-2019	28.59053	53.12299	[1]
Kh170-3	Khafr	*E. japonica*	1-October-2019	28.59053	53.12299	[1]
Kh170-4	Khafr	*E. japonica*	1-October-2019	28.59053	53.12299	[1]
Kh170-5	Khafr	*E. japonica*	1-October-2019	28.59053	53.12299	[1]
Kh170-6	Khafr	*E. japonica*	1-October-2019	28.59053	53.12299	[1]
Kh170-7	Khafr	*E. japonica*	1-October-2019	28.59053	53.12299	[1]
Gh093-1	Shiraz	*E. japonica*	1-October-2019	29.40582	52.28553	[1]
S43-s28	Bajgah	*Punica granatum*	26-November-2023	29.74111	52.58725	[3]
S41-s29	Bajgah	*P. granatum*	26-November-2023	29.74111	52.58725	[3]
S4-s51	Bajgah	*P. granatum*	26-November-2023	29.74111	52.58725	[3]
S4-s52	Bajgah	*P. granatum*	26-November-2023	29.74111	52.58725	[3]
S4-s53	Bajgah	*P. granatum*	26-November-2023	29.74111	52.58725	[3]
S4-s54	Bajgah	*P. granatum*	26-November-2023	29.74111	52.58725	[3]
S4-s55	Bajgah	*P. granatum*	26-November-2023	29.74111	52.58725	[3]
S4-s56	Bajgah	*P. granatum*	26-November-2023	29.74111	52.58725	[3]
S4-s57	Bajgah	*P. granatum*	26-November-2023	29.74111	52.58725	[3]
S12-s43	Bajgah	*P. granatum*	26-November-2023	29.74093	52.58724	[3]
S12-s45	Bajgah	*P. granatum*	26-November-2023	29.74093	52.58724	[3]
S14-s47	Bajgah	*P. granatum*	26-November-2023	29.74086	52.58724	[3]
S14-s48	Bajgah	*P. granatum*	26-November-2023	29.74086	52.58724	[3]
QW6-1	Qalat	*Juglans regia*	30-October-2023	29.81815	52.32269	[4]
QW6-2	Qalat	*J. regia*	30-October-2023	29.81818	52.32267	[4]
QW6-3	Qalat	*J. regia*	30-October-2023	29.81818	52.32263	[4]
QW6-4	Qalat	*J. regia*	30-October-2023	29.81818	52.32260	[4]
QW6-5	Qalat	*J. regia*	30-October-2023	29.81817	52.32269	[4]
QW6-6	Qalat	*J. regia*	30-October-2023	29.81817	52.32269	[4]
QW6-7	Qalat	*J. regia*	30-October-2023	29.81821	52.32261	[4]
QW6-8	Qalat	*J. regia*	30-October-2023	29.81821	52.32269	[4]
QW6-9	Qalat	*J. regia*	30-October-2023	29.81821	52.32268	[4]
QW6-10	Qalat	*J. regia*	30-October-2023	29.81818	52.32269	[4]
QW6-11	Qalat	*J. regia*	30-October-2023	29.81818	52.32271	[4]
QW6-12	Qalat	*J. regia*	30-October-2023	29.81816	52.32276	[4]
QW6-13	Qalat	*J. regia*	30-October-2023	29.81816	52.32265	[4]
QW8-1	Qalat	*J. regia*	30-October-2023	20.81802	52.32285	[4]
QW8-2	Qalat	*J. regia*	30-October-2023	20.81811	52.32291	[4]

* ex-type isolate (CBS 148864).

**Table 2:** Inter-simple sequence repeat (ISSR) primers used for the genetic diversity assessment of *Stilbocrea banihashemiana* isolates.

Primer Name	Sequence	Annealing Temperature (°C)	Total No. of Bands	Rang of Bands (bp)	No. of Polymorphic Bands	Polymorphism (%)	Reference
**M1**	5′-(ACTG)_4_-3′	49	25	123–2300	21	84	[26]
**M2**	5′-(GACAC)_4_-3′	45	17	138–2230	12	70	[26]
**M6**	5′-(GCC)_5_-3′	66	21	101–3150	17	80	[27]
**P5**	5′-(ACTG)_3_ACG-3′	46	20	195–2400	18	90	[28]
**P7**	5′-(TGTC)_5_-3′	45	13	220–2650	11	84.6	[29]
**P10**	5′-(GACC)_4_-3′	63	21	148–2890	12	57.1	[30]
**P17**	5′-(GGAGA)_3_-3′	45	16	162–2200	11	68.7	[31]
**PCMS**	5′-(GTC)_7_-3′	52	15	129–1614	12	80	[26]

**Table 3:** Indices for assessing the discriminating power of ISSR markers.

Primer	*PPL*	*I*	*H*	*PIC*	*He*	*Ho*	*K_e_*	*A*	*EMR*	*MI*
**M1**	33.54	0.587	0.402	0.404	0.409	0.404	1.70	2	49.64	19.45
**M2**	48.44	0.429	0.271	0.273	0.276	0.273	1.43	2	71.70	18.14
**M6**	39.09	0.511	0.340	0.354	0.358	0.354	1.59	2	57.85	19.33
**P5**	32.87	0.519	0.350	0.349	0.353	0.349	1.68	2	48.65	16.21
**P7**	33.78	0.533	0.355	0.356	0.360	0.365	1.59	2	50.00	18.74
**P10**	46.94	0.416	0.264	0.243	0.246	0.243	1.38	2	69.47	15.87
**P17**	46.03	0.464	0.308	0.306	0.310	0.306	1.52	2	68.12	18.75
**PCMS**	35.72	0.548	0.370	0.371	0.375	0.371	1.65	2	52.86	18.38
**Mean**	39.55	0.500	0.332	0.332	0.335	0.333	1.56	2	58.54	18.11

*A*: Allele number, *EMR*: Effective multiplex ratio, *He*: Expected heterozygosis, *Ho*: Observed heterozygosis, *H:* Nei’s gene diversity index, *I*: Shannon index, *K_e_*: Effective allele number, *MI*: Marker index, *PIC*: Polymorphic information content, *PPL*: Percentage of polymorphic loci.

## Data Availability

The original contributions presented in this study are included in the article/Supplementary Materials. Further inquiries can be directed to the corresponding authors.

## Supplementary Materials

The following supporting information can be downloaded at: https://www.mdpi.com/article/10.3390/plants15131945/s1, Figure S1. Cultural and asexual morphological characteristics of *Stilbocrea banihashemiana* from infected fig trees in central regions of Fars Province, Iran. Colonies on PDA after 14 days at 25 °C under a 12 h photoperiod, obverse and reverse side view, respectively (a, b); Abundant globose conidia produced by phialides and conidiophores (c); Smooth, hyaline, and unicellular conidia, in two different forms globose and allantoid (d). (Bar = 10 μm). Figure S2. Geographic distribution of *Stilbocrea banihashemiana* isolates recovered from various cultivars of *Ficus carica* in Fars Province, Iran, during a five-year (2019–2023) survey. Circles size is proportional to the number of isolates obtained per location. Figure S3. Species-specific PCR identification of *Stilboarea banihashemiana* isolates from infected fig trees in Fars Province. Amplification was performed using primer pairs (a) TEF Sb1 (TEF SbF1/TEF SbR1), yielding a 443 bp product, and (b) TEF Sb3 (TEF SbF1/TEF SbR2), yielding a 577 bp product. Lanes 1–38 correspond to the isolates collected in this study. M: 100 bp DNA ladder (with selected bands labeled at 500 bp, 1500 bp, and 3000 bp). Lane 39: negative control (no template DNA).; Table S1. Analysis of variance (ANOVA) of four pathogenicity characteristics induced by *Stilbocrea banihashemiana* isolates (n =53) on detached shoots of fig trees; Table S2. Mean values of four pathogenicity traits induced by 53 *Stilbocrea banihashemiana* isolates in a detached fig shoot assay. Within each column, means followed by different letters differ significantly (*p* ≤ 0.05). Table S3. Analysis of variance (ANOVA) of internal lesion length and width induced by *Stilbocrea banihashemiana* (ex-type, isolate FS1 = CBS 148864) on saplings of 10 fig cultivars; Table S4. Specific primer pairs for *Stilbocrea banihashemiana* based on the *tef1* gene used in this study [49].

## Abbreviations

The following abbreviations are used in this manuscript:

*A*	Number of alleles
ANOVA	Analysis of variance
CRD	Completely randomized design
CV	Coefficient of variation
df	Degrees of freedom
DILP	Downward internal lesion progression
*EMR*	Effective multiplex ratio
GLM	General linear model
*H_e_*	Expected heterozygosity
HSD	Honestly Significant Difference
*H*	Nei’s diversity index
*H_o_*	Observed heterozygosity
ISSR	Inter-simple sequence repeat
ILL	Internal lesion length
ILW	Internal lesion width
*I*	Shannon index
*K_e_*	Effective allele number
MS	Mean square
*MB*	Monomorphic bands
*MI*	Marker index
*PPL*	Percentage of polymorphic loci
*PIC*	Polymorphism information content
*PB*	Polymorphic bands
*PB*	Potato broth
PCA	Principal component analysis
PCoA	Principal coordinate analysis
PCR	Polymerase chain reaction
PDA	Potato dextrose agar
PR	Pathogenesis-related
S.O.V.	Source of variation
*tef1*	Translation elongation factor 1-α
UPGMA	Unweighted pair group method with arithmetic averages
UILP	Upward internal lesion progression
VP	Vascular progression
WA	Water Agar
