# Supplementary material for: Genetic Variability and Aggressiveness of Stilbocrea banihashemiana, an Emerging Pathogen Responsible for Cankers of Fig and Fruit Trees
Source: Plants (Basel). 2026 Jun 24;15(13):1945. doi: 10.3390/plants15131945 (PMC13364062; doi:10.3390/plants15131945)
Supplement: Supplementary file 1 [file plants-15-01945-s001.zip › plants-4311899-supplementary.pdf]

Table S1. Analysis of variance (ANOVA) of four pathogenicity characteristics induced by *Stilbocrea banihashemiana* isolates (n =53) on detached shoots of fig trees.

S.O.V	Df	Lesion length			Lesion width			Upward internal lesion progression			Upward internal lesion progression		
		MS	F-value	*P*-value	MS	F-value	*P*-value	MS	F-value	*P*-value	MS	F-value	*P*-value
**Isolate**	52	144.41	46.86	<.0001	6.25	8.95	<.0001	239.23	50.96	<.0001	111.26	75.94	<.0001
**Error**	212	3.08			0.70			4.69			1.46		
**CV (%)**			10.69			9.85			12.46			10.99	

Note: S.O.V: Source of variation; Df: Degrees of freedom; MS: Mean square, CV: Coefficient of variation.

Table S2. Mean values of four pathogenicity traits induced by 53 *Stilbocrea banihashemiana* isolates in a detached fig shoot assay. Within each column, means followed by different letters differ significantly ($P \leq 0.05$).

Isolate code	Lesion length *	Lesion width	Upward internal lesion progression	Downward internal lesion progression
**ECH218-1**	15.2^{h-n} ± 1.3	8.6^{b-f} ± 0.9	13.0^{m-u} ± 1.2	10.6^{i-n} ± 0.9
**EK234-1**	11.4^{nop} ± 0.5	7.0^{ef} ± 0.7	10.4^{q-v} ± 0.9	7.4^{op} ± 0.5
**EK234-2**	12.8^{j-p} ± 1.3	7.4^{def} ± 0.5	14.4^{k-q} ± 0.9	10.2^{j-o} ± 0.8
**ES227-1**	12.2^{k-p} ± 1.6	7.6^{def} ± 1.0	18.0^{g-m} ± 0.5	8.4^{l-p} ± 3.5
**ES227-2**	16.6^{g-k} ± 0.9	8.4^{b-f} ± 0.5	12.4^{n-v} ± 1.4	10.0^{j-p} ± 1.2
**ES227-3**	14.6^{h-p} ± 1.1	8.4^{b-f} ± 0.6	13.2^{m-u} ± 1.3	7.6^{nop} ± 0.5
**ES227-4**	12.0^{l-p} ± 0.7	8.0^{b-f} ± 0.7	14.4^{k-q} ± 1.7	9.2^{k-p} ± 1.3
**ES229-1**	16.4^{g-l} ± 2.1	8.5^{b-f} ± 1.4	17.4^{g-n} ± 1.7	13.6^{d-h} ± 1.5
**ES229-2**	10.8^{nop} ± 0.8	8.0^{b-f} ± 0.7	10.8^{p-v} ± 1.3	8.2^{m-p} ± 0.8
**ES229-3**	17.6^{f-i} ± 2.1	7.6^{def} ± 0.5	14.6^{k-q} ± 1.3	7.4^{op} ± 0.5
**ES229-4**	12.0^{l-p} ± 0.7	7.6^{def} ± 0.9	13.8^{l-t} ± 2.0	7.6^{nop} ± 0.9
**ES229-5**	15.8^{h-o} ± 2.4	9.4^{bcd} ± 1.1	17.0^{g-o} ± 2.7	8.4^{l-p} ± 1.6
**ES229-6**	19.1^{e-h} ± 1.0	8.8^{b-f} ± 0.8	17.4^{g-n} ± 1.3	10.6^{i-n} ± 1.7
**ES229-8**	10.8^{nop} ± 1.1	8.4^{b-f} ± 1.1	8.4^{tuv} ± 1.1	7.0^{p} ± 0.7
**ES231-1**	11.8^{m-p} ± 1.0	7.6^{def} ± 0.7	10.8^{p-v} ± 1.7	7.4^{op} ± 1.1
**ES231-1-1**	12.8^{j-p} ± 1.1	7.5^{def} ± 0.5	14.0^{k-s} ± 1.0	8.6^{l-p} ± 0.9
**ES231-2**	16.0^{g-m} ± 1.0	8.0^{b-f} ± 0.7	11.6^{o-v} ± 1.7	9.8^{j-p} ± 1.1
**ES231-4**	12.1^{k-p} ± 0.6	9.1^{b-f} ± 0.9	19.0^{g-l} ± 2.3	8.4^{l-p} ± 1.5
**ES231-5**	12.0^{l-p} ± 1.2	7.7^{c-f} ± 0.8	14.8^{k-q} ± 0.8	7.8^{m-p} ± 0.8
**ES231-5-1**	21.2^{def} ± 0.8	9.4^{bcd} ± 0.5	19.4^{g-k} ± 2.2	10.4^{i-p} ± 1.7
**ES231-6**	10.6^{op} ± 0.5	7.6^{def} ± 0.9	8.7^{s-v} ± 0.8	7.8^{m-p} ± 1.3
**ES232-2**	23.4^{cde} ± 2.4	9.0^{b-f} ± 1.0	16.8^{g-o} ± 1.3	15.0^{def} ± 1.0
**ES232-2-2**	13.0^{i-p} ± 0.7	7.4^{def} ± 0.5	13.4^{g-o} ± 1.5	12.0^{f-k} ± 0.7
**ES232-2-3**	14.6^{h-p} ± 1.7	8.0^{b-f} ± 0.7	18.4^{g-m} ± 1.1	9.4^{k-p} ± 1.1
**ES232-2-8**	12.8^{j-p} ± 1.1	7.4^{def} ± 0.5	13.8^{l-t} ± 1.7	11.4^{i-l} ± 0.5
**ES232-3**	11.0^{nop} ± 1.2	7.5^{def} ± 0.5	8.2^{uv} ± 1.3	7.8^{m-p} ± 0.8
**ES232-4**	13.6^{i-p} ± 1.5	7.4^{def} ± 0.6	15.8^{i-q} ± 1.3	12.6^{e-j} ± 0.9

Isolate code	Lesion length	Lesion width	Upward internal lesion progression	Downward internal lesion progression
**ES235-1**	17.0^{f-j} ± 2.0	9.0^{b-f} ± 0.7	19.2^{g-l} ± 2.3	8.2^{m-p} ± 0.8
**ES235-2**	10.8^{nop} ± 0.8	7.2^{ef} ± 0.4	10.2^{r-v} ± 1.5	7.0^{p} ± 0.7
**Esi186-1**	29.2^{a} ± 2.3	11.9^{a} ± 0.8	31.8^{bc} ± 3.1	23.2^{bc} ± 2.1
**FS1**	12.8^{j-p} ± 2.0	8.6^{b-f} ± 0.5	14.4^{k-q} ± 2.1	8.6^{l-p} ± 1.1
**Gh093-1**	13.0^{j-p} ± 1.4	7.4^{def} ± 0.5	11.0^{p-v} ± 1.2	7.6^{nop} ± 0.9
**GhSa11-1**	23.4^{cde} ± 3.8	9.4^{bcd} ± 0.5	20.8^{f-j} ± 2.9	15.2^{de} ± 1.3
**GhSa11-2**	15.0^{h-o} ± 1.0	9.8^{abc} ± 0.8	29.8^{bcd} ± 3.0	13.4^{d-i} ± 1.9
**GhSa11-5-1**	16.0^{g-m} ± 1.6	8.0^{b-f} ± 0.7	19.2^{g-l} ± 2.6	13.4^{d-i} ± 1.1
**Kh170-1**	15.2^{h-n} ± 0.8	7.8^{c-f} ± 0.8	15.4^{j-r} ± 1.3	11.4^{i-l} ± 0.5
**Kh170-2**	16.6^{g-k} ± 3.1	8.2^{b-f} ± 1.1	15.6^{i-r} ± 2.3	10.0^{j-p} ± 1.9
**Kh170-4**	24.0^{bcd} ± 1.9	8.4^{b-f} ± 0.5	21.9^{efg} ± 1.8	10.8^{i-m} ± 0.8
**Kh170-6**	12.7^{j-p} ± 0.8	9.4^{bcd} ± 1.1	27.0^{cde} ± 7.2	8.8^{l-p} ± 1.3
**Kh170-7**	10.4^{p} ± 0.5	7.4^{def} ± 0.5	14.2^{k-s} ± 1.5	7.7^{m-p} ± 0.7
**NS195-1**	28.4^{ab} ± 1.8	11.6^{a} ± 0.6	34.8^{ab} ± 1.3	25.4^{b} ± 1.1
**NS199-1**	21.2^{def} ± 1.9	8.0^{b-f} ± 0.7	15.6^{i-r} ± 1.7	10.6^{i-n} ± 1.3
**NS202**	29.6^{a} ± 3.4	11.8^{a} ± 0.8	39.6^{a} ± 1.1	29.4^{a} ± 2.3
**NSDrj-1**	10.2^{p} ± 0.8	7.0^{ef} ± 0.7	7.0^{v} ± 0.7	7.4^{op} ± 0.5
**QW6-1**	17.8^{f-i} ± 1.5	8.6^{b-f} ± 0.5	21.4^{fgh} ± 1.9	10.6^{i-n} ± 0.9
**QW6-10**	13.4^{i-p} ± 1.5	9.4^{bcd} ± 1.1	13.8^{l-t} ± 2.0	7.6^{nop} ± 0.5
**QW8-1**	27.4^{abc} ± 1.8	10.0^{ab} ± 1.2	29.6^{bcd} ± 3.1	14.0^{d-g} ± 2.0
**QW8-2**	16.4^{g-l} ± 3.2	8.8^{b-f} ± 1.1	21.2^{f-i} ± 2.2	7.8^{m-p} ± 0.8
**S12-s43**	19.8^{d-g} ± 2.8	9.2^{b-e} ± 1.8	26.2^{def} ± 1.9	16.2^{d} ± 1.9
**S14-s47**	23.6^{cd} ± 2.1	8.6^{b-f} ± 0.5	21.4^{fgh} ± 3.8	14.0^{d-g} ± 1.2
**S41-s29**	17.6^{f-i} ± 1.5	8.2^{b-f} ± 1.3	16.2^{h-p} ± 1.5	9.8^{j-p} ± 1.1
**S43-s28**	20.2^{d-g} ± 3.2	9.0^{b-f} ± 1.2	16.2^{h-p} ± 2.7	9.4^{k-p} ± 1.3
**S4-s51**	27.2^{abc} ± 2.6	9.8^{abc} ± 0.8	28.0^{cd} ± 2.0	21.6^{c} ± 1.5

*mm; mean ± standard deviation (n = 5).

Table S3. Analysis of variance (ANOVA) of internal lesion length and width induced by *Stilbocrea banihashemiana* (ex-type, isolate FS1= CBS 148864) on saplings of 10 fig cultivars.

S.O.V	Df	Internal lesion length			Internal lesion width		
		MS	F-value	*P*-value	MS	F-value	*P*-value
**Cultivar**	9	24.80	11.46	<.0001	6.84	8.79	<.0001
**Error**	20	2.16			0.79		
**CV (%)**			17.12			10.89	

Note: S.O.V: Source of variation; Df: Degrees of freedom; MS: Mean square, CV: Coefficient of variation.

Table S4. Specific primer pairs for *Stilbocrea banihashemiana* based on the *tef1* gene used in this study (Negahban *et al*. 2024a).

Primer pairs	Primer	Product length (bp)	Sequence (5′ to 3′)	Target (GenBank accession no.)	Location
**TEF-Sb1**					
	TEF-SbF1	443	GCCTTATCTGCTCTGGTGGGG	OM876865	71-514
	TEF-SbR1		GAGGGTGTAAGCGAGCAGAG		
**TEF-Sb3**					
	TEF-SbF1	577	GCCTTATCTGCTCTGGTGGGG	OM876865	71-648
	TEF-SbR2		AAAGGAACGGTCTTGGGGTT		

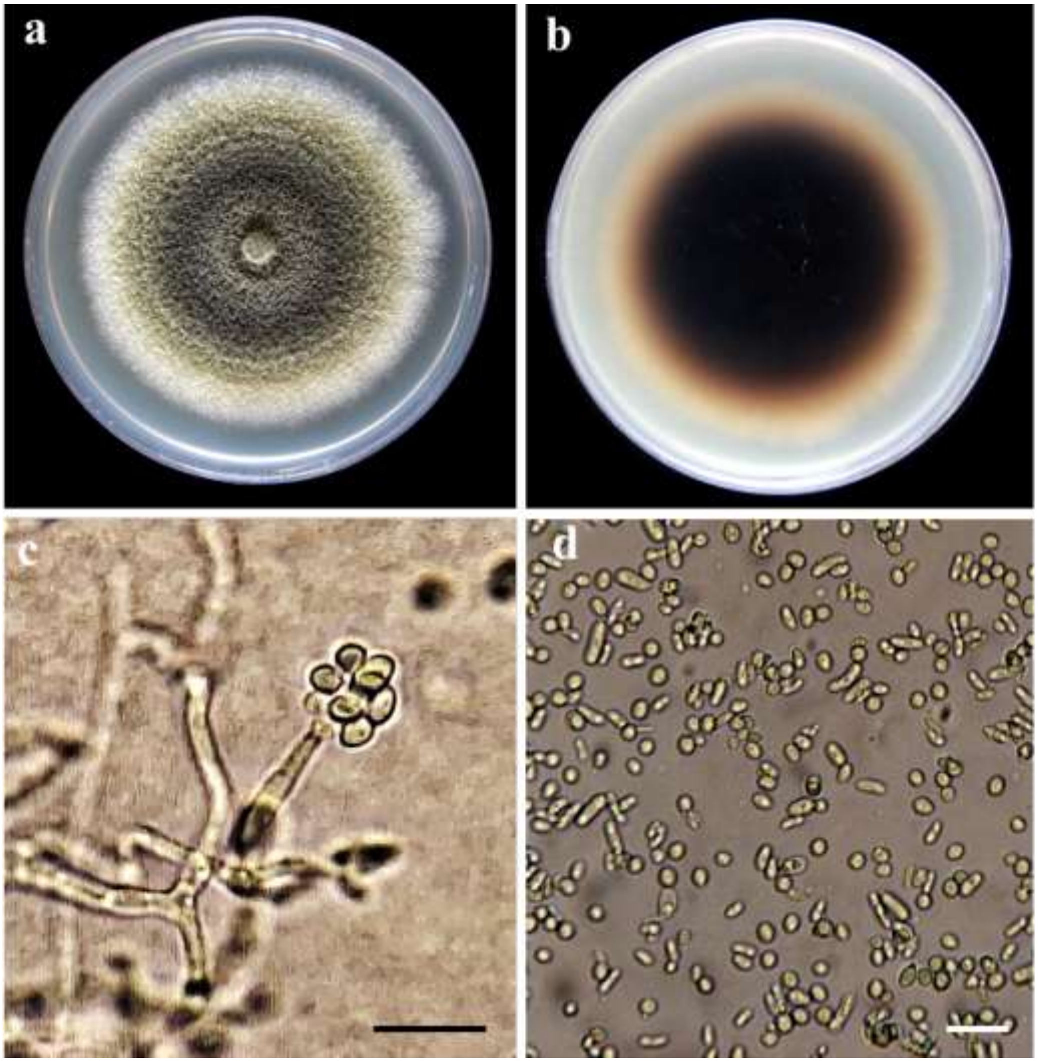

Figure S1. Cultural and asexual morphological characteristics of *Stilbocrea banihashemiana* from infected fig trees in central regions of Fars Province, Iran. Colonies on PDA after 14 days at 25 °C under a 12-h photoperiod, obverse and reverse side view, respectively (a, b); Abundant globose conidia produced by phialides and conidiophores (c); Smooth, hyaline, and unicellular conidia, in two different forms globose and allantoid (d). (Bar = 10 μm).

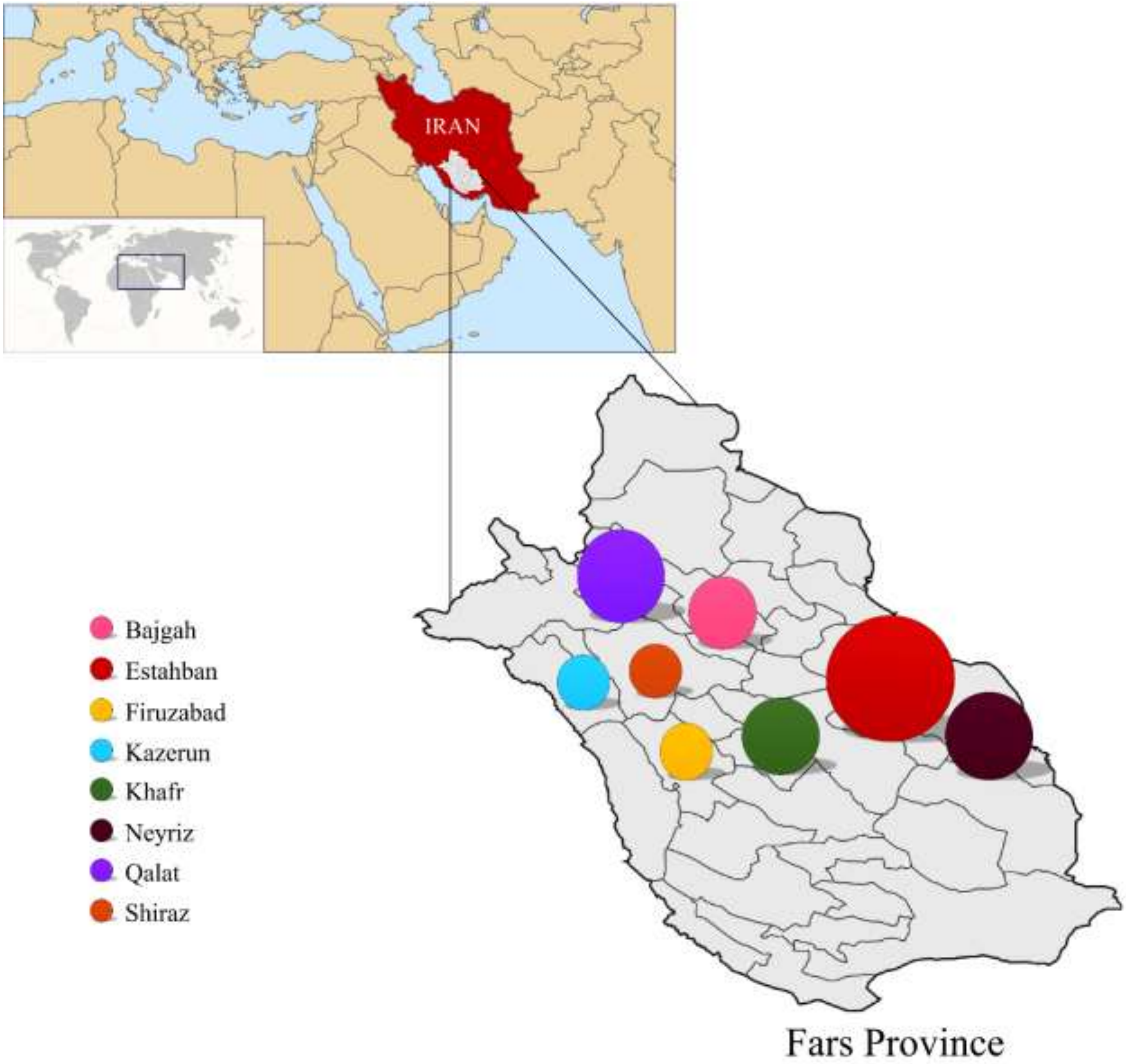

Figure S2. Geographic distribution of *Stilbocrea banihashemiana* isolates recovered from various cultivars of *Ficus carica* in Fars Province, Iran, during a five-year (2019-2023) survey. Circles size is proportional to the number of isolates obtained per location.

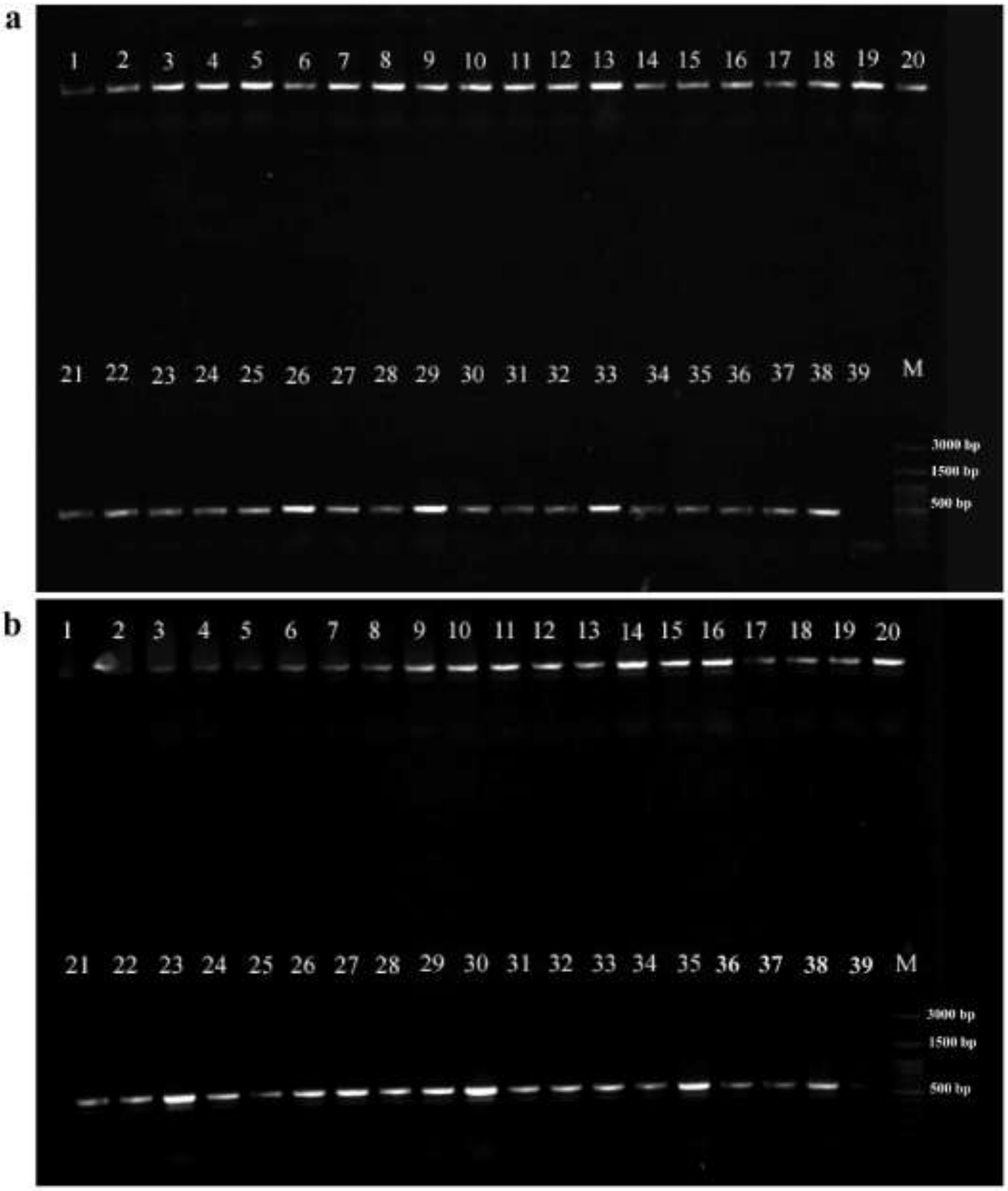

Figure S3. Species-specific PCR identification of *Stilboarea banihashemiana* isolates from infected fig trees in Fars Province. Amplification was performed using primer pairs (a) TEF-Sb1 (TEF-SbF1/TEF-SbR1), yielding a 443 bp product, and (b) TEF-Sb3 (TEF-SbF1/TEF-SbR2), yielding a 577 bp product. Lanes 1–38 correspond to the isolates collected in this study. M: 100 bp DNA ladder (with selected bands labeled at 500 bp, 1500 bp, and 3000 bp). Lane 39: negative control (no template DNA).
